# Effects of stigmatizing media coverage on stigma measures, self-esteem, and affectivity in persons with depression – an experimental controlled trial

**DOI:** 10.1186/s12888-019-2123-6

**Published:** 2019-05-07

**Authors:** Nele Cornelia Goepfert, Steffen Conrad von Heydendorff, Harald Dreßing, Josef Bailer

**Affiliations:** 1 Department of Clinical Psychology, Central Institute of Mental Health, Medical Faculty Mannheim, University Heidelberg, J5, 68159 Mannheim, Germany; 2 Department of Psychiatry and Psychotherapy, Central Institute of Mental Health, Medical Faculty Mannheim, University Heidelberg, J5, 68159 Mannheim, Germany; 3 Department of Forensic Psychiatry, Central Institute of Mental Health, Medical Faculty Mannheim, University Heidelberg, J5, 68159 Mannheim, Germany

**Keywords:** Stigma, Self-stigma, Depression, Media

## Abstract

**Background:**

Stigmatization of people with mental illness is still a significant problem even in Western society. Media is an important vector for public messaging that may lead to stigma (and potentially counteract it). There is an ongoing debate about the impact of news with potentially stigmatizing content on people with depression. This experimental study aimed at investigating the direct effects media reporting could have on people with depression, namely, higher levels of stigma attitudes and negative affect, as well as lower levels of self-esteem and positive affect.

**Methods:**

Experimental study; target sample size *n* = 180 patients; eligibility criteria: clinical diagnosis of depressive episode or dysthymia, aged 18–70 years, sufficient cognitive abilities and German language skills; exclusion criteria: acute psychotic, manic or hypomanic episode, addiction symptoms, or suicidal ideation; parallel assignment to one of three arms (each *n* = 60): watching a short film about a negative event relating to depression (experimental group), about a negative event without relation to depression (control group 1), or about a neutral event relating to depression (control group 2); primary outcomes: degrees of stigma attitudes (stereotype awareness, stereotype agreement, self-concurrence, and self-stigmatization); secondary outcomes: degrees of self-esteem, positive and negative affect; statistical analyses: general linear models with repeated-measures; one-way ANOVAs of the change in scores, followed by Bonferroni-adjusted pairwise comparisons; IBM SPSS Statistics 24.0.

**Results:**

Significant group × time interactions in stereotype agreement (medium effect: *η =* 0.10) and negative affect (large effect: *η =* 0.26); the level of stereotype agreement increased significantly more in the experimental group than in control groups 1 and 2. The level of negative affect increased significantly more in the experimental group and in control group 1 than in control group 2. All other interaction effects were non-significant.

**Conclusion:**

The present study allows statements about the direct effects of potentially stigmatizing media reporting on carriers of the stigmatized attribute, i.e., depression: Even single film presentations of familiar events that contain potentially stigmatizing content have an impact on stereotype agreement and negative affect. The impact of long-term exposure and change in other stigma-measures require a deeper understanding of stigma-processes. Potential explanations and implications for practice and future research are discussed.

**Trial registration:**

Deutsche Register Klinischer Studien, Trial registration: DRKS00011855. Registered 23 June 2017, retrospectively registered; for details see Additional file 1.

**Electronic supplementary material:**

The online version of this article (10.1186/s12888-019-2123-6) contains supplementary material, which is available to authorized users.

## Background

Despite major efforts by regional and national educational programmes, the stigmatization of people with mental illness is still a significant problem even in Western society [1]. Goffman defines “stigma as a mark (attribute) that links a person to undesirable characteristics (stereotypes)” [2]. Following this definition, media is an important vector for public messaging that may lead to stigma (and potentially counteract it) [3–5]. Several overviews of mental illness in both fictional and nonfictional media and its complex role in the context of depression and suicidality can be found in the literature [6–8]. In addition to factual information, mental illness is frequently reported in the context of exciting incidents [9], linking mental illness to “danger”, “crime”, and a “negative burden on society” [10]. As a consequence, collective stigmatizing assumptions about persons with mental illness (public stigmatization) are reinforced.

### Media and public stigmatization

Even in cases of uncertainty whether a negative event was caused by human or technical failure, associations are quickly drawn with potential involvement of mental illness in media coverage. The March 2015 Germanwings plane crash in France generated particularly extensive news coverage with potentially stigmatizing content about depression [11].

Many experts criticized such potentially stigmatizing media coverage (SMC) in the case of the plane crash, postulating higher levels of public stigmatization and self-stigmatization as a consequence (e.g., [12, 13]). To date, there is little empirical evidence on which these public discussions regarding effects of SMC about events related to depression could be sufficiently based. Corrigan et al. found both positive and negative effects of news stories regarding mental illness [14]. Priming audiences about mental illness in general by just mentioning mental illness in the context of violent incidents makes them infer a causal link between them [15]. In the early 1990s, when Oskar Lafontaine and Wolfgang Schäuble, two popular German politicians, were attacked by perpetrators with psychoses, public stigmatization significantly increased in the German population [16]. The overall increase in the community’s stigma attitudes towards persons with depression between 2014 and 2015 after the Germanwings plane crash was smaller than postulated [17]: Only a few significant changes were indicated in the perceived separation of persons afflicted and stereotypes on item level (more unpredictable, less in need of help), and marginally in emotional reactions (anger, fear).

### Self-stigmatization

Direct effects that SMC could have on those suffering from specific stigmatized mental disorders have not yet been sufficiently studied. Corrigan and Watson added the stigmatized group’s perspective to Goffman‘s model of stigma [18]. A process model of four succeeding stages was postulated during which perceived public stigma results in self-stigmatization in persons who carry the stigmatized attribute (i.e., internalization of stigma experience) [19]: stereotype awareness (perception of public stigma), followed by stereotype agreement (believing public stigma to be true), self-concurrence (internalizing stereotypes and applying them to oneself), and harm-to-self (e.g., lower levels of self-esteem). Therefore, even if there was no definite effect of SMC on the general public’s stigma in the case of the plane crash, stereotype awareness and stereotype agreement may have been increased in persons with depression followed by self-stigmatization and harm-to-self. A higher level of self-stigmatization in turn is negatively related to well-being [20], quality of life, professional help-seeking when needed [21], general performance, self-esteem [22], self-clarity, hope, recovery, and functioning [23], and positively related to suicidal behaviour [24].

### Objective and hypotheses

The aim of this study was to shed light on direct effects of SMC on stereotype awareness, stereotype agreement, self-concurrence, and harm-to-self.

It could be expected that watching potentially stigmatizing media reports would increase the levels of stereotype awareness, stereotype agreement, self-concurrence and negative affect in persons with the stigmatized attribute, i.e., depression. At the same time, watching stigmatizing media reports would decrease the level of self-esteem and positive affect.

To control for stigma-specific content effects and affectivity effects, three groups were compared in this study. The experimental group (EG) watched a film that was both about a *negative event* and *referring to depression*. Control group 1 (CG1) watched a film about a *negative event* but *without reference to depression*. While a more negative affect could be expected after the groups watched the films, an effect on stigma-measures was expected in EG only. Control group 2 (CG2) watched a film that was about a *neutral event* and *referring to depression*. The effect on affectivity was expected to be smaller than in EG and CG1; the effect on stigma-measures was expected to be the reverse of that with EG but not significantly different from CG1.

## Methods

The study was conducted as an experimental laboratory trial using a controlled design with parallel randomized groups, comparing three different conditions. Ethical approval to conduct the study was received from the Ethical Committee of the Medical Faculty of Mannheim, Heidelberg University, Germany (study ID 2016-655 N-MA). The study is registered in German Clinical Trials Register (for details see Additional file 1).

Considering clinical relevance and therefore expecting a small to medium effect size, target sample size was estimated using G*power 3.1 indicating a target sample size of *n* = 177 (power = 0.95, α = 0.05, effect size(f) = 0.15, 3 groups, 2 measurements) for variance analyses with repeated measures (within-between interaction).

### Participants

**Fig. 1:**
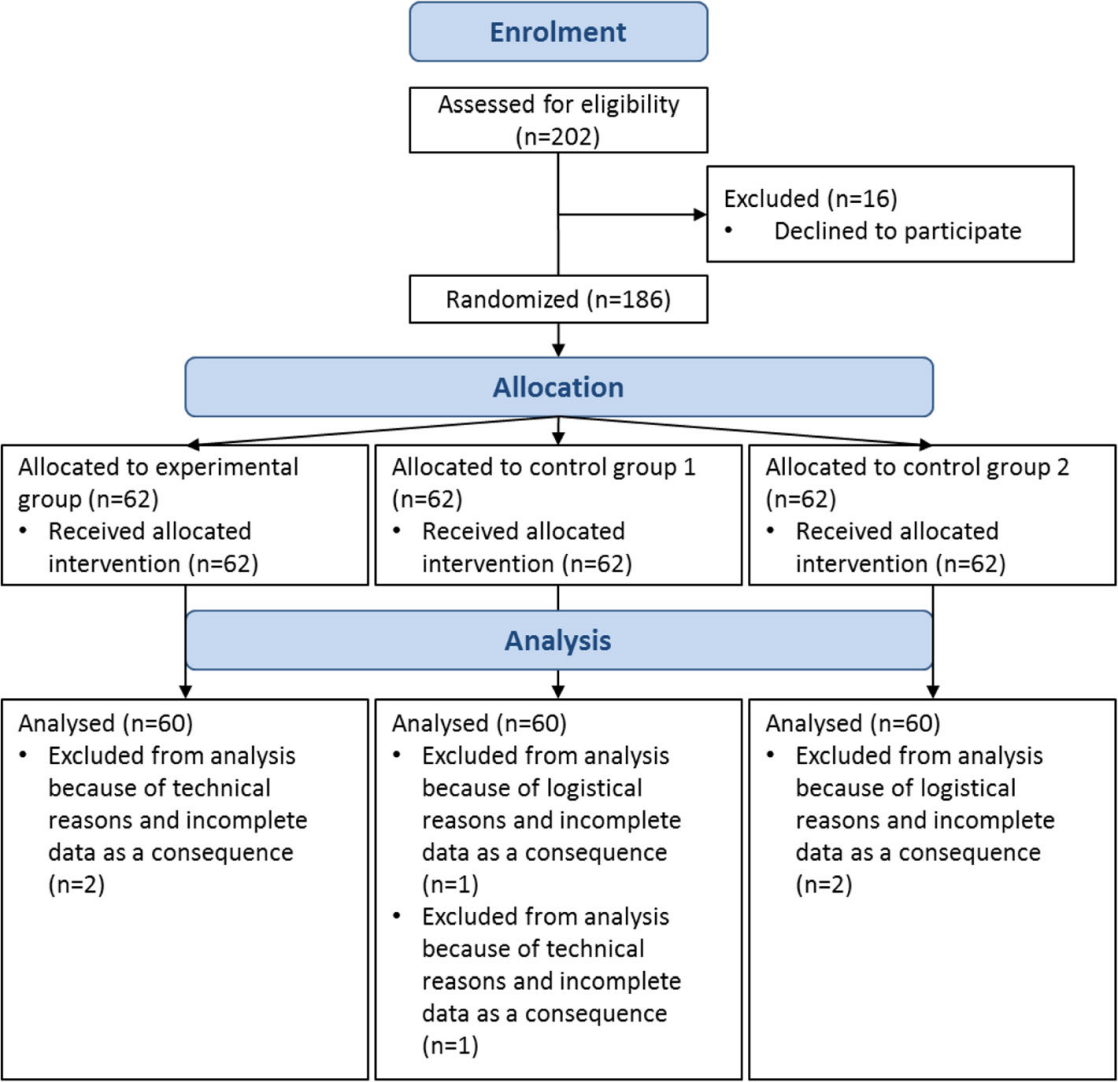
Progress through the phases of the parallel randomized trial of three groups

Participants were recruited from the Central Institute of Mental Health in Mannheim (CIMH) in Germany by their treating doctors and psychotherapists from 03/2017 to 07/2018. Eligibility criteria for participants were at least one pre-diagnosed depressive episode or dysthymia, age of 18–70 years, sufficient cognitive abilities and German language skills. Exclusion criteria were acute psychotic, manic or hypomanic episodes; addiction symptoms; or acute suicidal ideation. Patients who were assumed to lack capacity to freely provide informed consent were excluded by the treatment staff in advance. Half of the participants were outpatients and the other half were inpatients treated at the CIMH. The study consisted of two parts, i.e., a screening interview and an experimental phase (for details please refer to section procedure). Two hundred two persons went through the screening interview. Of those, one hundred eighty-six patients could be recruited for study participation. For technical and logistical reasons, 6 incomplete data sets had to be excluded from analyses. The study progress is presented in Fig. 1.

### Procedure

During a standardized screening interview, eligibility and exclusion criteria were examined and oral informed consent was given. An individual code was generated. Personal information was documented separately from screening data to guarantee confidentiality after enrolment. The code was used only to merge anonymous data.

Before participation in the experimental phase, informed consent was ensured by detailed written forms signed by all participants. Participants received a 20€ expense allowance. Data collection and film presentations were computer-based. Participants filled in a baseline questionnaire and rated their current level of positive and negative affect. By viewing a short film about the bird of the year 2016 (nature documentary about woodpeckers; 2 min and 40 s), participants could become familiar with the laboratory setting and the method applied.

Positive and negative affect, level of valence, arousal, and familiarity of the film were assessed for manipulation check. Participants were then randomly assigned to one of three conditions. An allocation sequence list was based on computer-generated random numbers before recruitment. Experimenters allocated participants to conditions chronologically, corresponding to the sequentially numbered allocation list, which was generated with Excel prior to recruitment.

#### Conditions

##### Experimental group (negative event relating to depression)

Participants in the experimental group watched a short film (4 min and 21 s) that was based on news about the 2015 Germanwings plane crash in France retrieved from a database of www.ARD.de, a public TV channel. Several reports had been scanned and essential parts had been cut together to keep the testing time within an acceptable range whil still covering essential information. In the film, reporters and representatives of Germanwings give statements about the pilot’s clinical diagnosis of depression as a main reason for the incident. They call for more transparency and access to employees’ medical records. This condition therefore covered both reporting about an exciting negative event and linking it to clinical diagnoses of depression.

##### Control group 1 (negative event without relation to depression)

Participants in CG1 watched a film (4 min and 7 s) about news regarding the Fukushima catastrophe in March 2011 and its possible consequences for Japan and the world. The source was the same as that of the film of the experimental condition. Although this condition concerns an exciting negative event, no reference is made to mental illness. It can therefore control for negative affect, which is not related to stigmatization.

##### Control group 2 (neutral event relating to depression)

Participants in CG2 watched a film (3 min and 56 s) about the second congress on depression organized by “Stiftung Deutsche Depressionshilfe” in cooperation with “Deutsches Bündnis gegen Depression” and “Deutsche Depressionsliga”. This documentary film neutrally reports on an event referring to depression but without any exciting negative incidents. It can therefore control for reactions that refer to depression but without any stigmatizing context. Relevant dependent variables were assessed immediately before and after the film presentations.

**Fig. 2:**
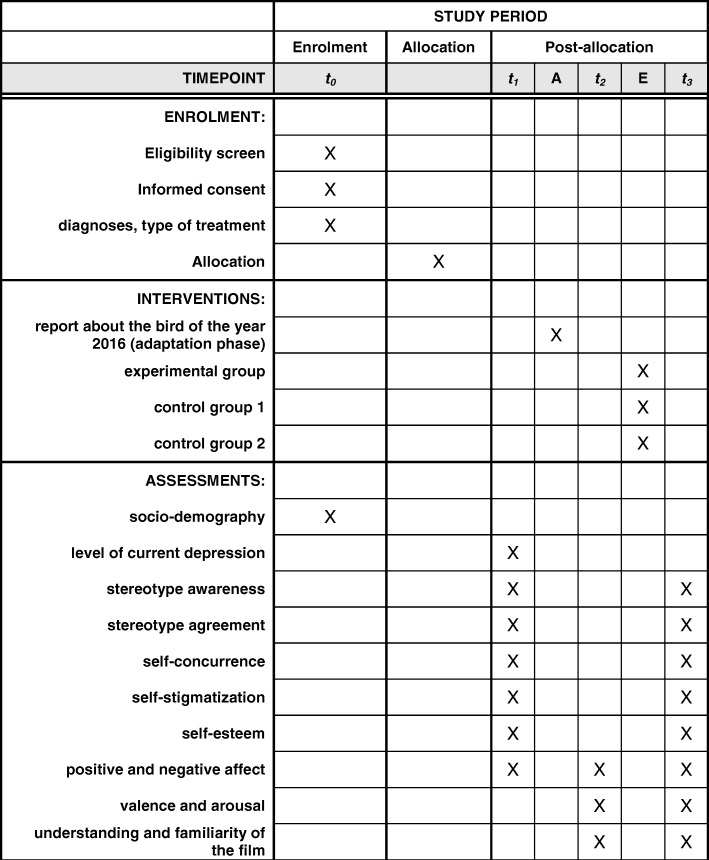
Schedule of enrolment, interventions, and assessments. Legend: A = Adaptation phase: A short nature documentary was presented to familiarize participants with the laboratory setting and the method applied; E = Experimental phase: Participants were randomly assigned to one of three conditions. Times of measurements: t_0_ = screening phase; t_1_ = baseline measurement before watching any films; t_2_ = after watching the nature documentary; t_3_ = after watching the second film

In sum, there were three times when measurements were taken in addition to screening: t_1_ = baseline measurement before watching any films; t_2_ = after watching the film about the bird of the year; t_3_ = after watching the second film. Please refer to Fig. 2 for schedule of enrolment, interventions, and assessments at specific points in time.

### Measures

#### Self-stigma

The development and psychometrics of the self-stigma of mental illness scale (SSMIS) were presented by Corrigan et al. [25], German version by Rüsch and Brück, published in [26]. Of the four subscales, containing 10 items each, the first three subscales were used in the current study, namely, stereotype awareness, stereotype agreement, and self-concurrence. Stereotype awareness refers to beliefs about the public’s attitudes towards people with mental illness; stereotype agreement covers one’s own beliefs about people with mental illness in general; self-concurrence implicates a causal relation between one’s mental illness and stereotype characteristics. Response scales ranged from 1=“I strongly disagree” to 9=“I strongly agree”. Cronbach’s alpha was α = 0.91 for stereotype awareness, α = 0.87 for stereotype agreement, and α = 0.81 for self-concurrence.

#### Self-esteem

Based on Watson et al.’s [27] experience, the fourth SSMIS subscale of self-esteem decrement was not included in this study because of its difficult wording. Instead, a revised German adaptation of the well-known Rosenberg’s self-esteem scale consisting of 10 items was used [28, 29]. Cronbach’s alpha was α = 0.87.

#### Self-stigma of depression

The German adaptation of the Self-Stigma of Depression Scale for people with depression (SSDS-D) was added for measuring sample specific self-stigma of depression in comparison to self-stigma of mental illness in general [30]. The original Self-Stigma of Depression Scale was the first scale to measure anticipated self-stigma in cases of mental illness, i.e., how people would feel or think if they had a depressive disorder [31]. The German adaptation SSDS-D covers actually experienced self-stigma of people with depression. It consists of 16 items on 4 subscales, namely, shame (3 items), self-blame (5 items), social inadequacy (4 items), and help-seeking inhibition (4 items). Patients were asked to what extent they agreed with self-stigmatising attitudes regarding their depressive disorder (example: “I feel ashamed about it.”). The response scale ranged from 1=“I do not agree at all” to 5=“I totally agree”. Cronbach’s alpha was α = 0.86 for shame, α = 0.82 for self-blame, α = 0.74 for social inadequacy, and α = 0.83 for help-seeking inhibition.

#### Positive and negative affect

The positive and negative affect scale consists of 2 subscales, namely, positive affect and negative affect, of 10 adjectives each (PANAS; [32]). Patients were asked to indicate how much they currently related to each adjective in terms of how they were feeling. The response scales ranged from 1=“not at all” to 5 = “extremely”. Cronbach’s alpha was α = 0.89 for positive and α = 0.86 for negative affect. The PANAS was used to test changes in affect state after viewing the films.

#### Socio-demographic and clinical characteristics

The current severity of depression symptoms has been shown to be highly correlated with the current level of self-esteem [33]. It was measured via the German version of the Patient Health Questionnaire [34, 35]. Internal consistency of the 9-item PHQ-depression scale was α = 0.84. Age in years and gender have been inconsistently correlated with self-stigma in previous research and are therefore also assessed [36].

#### Manipulation check

For manipulation check of the films, the videos were rated applying von Heydendorff’s and Dressing’s categorical system of critical coverage [11]. It measures aspects of media reports that imply a causal relationship between negative events and mental illness. Additionally, patients rated on a scale from 1=“not at all” to 9 = “extremely” the level of familiarity and arousal as well as valence on a 9-point scale from 1 = “very negative” to 9 = “very positive”.

### Data management and statistical methods

Data collection was online based via SoSci Survey, a professional tool for online surveys. As such, data could be directly exported to IBM SPSS Statistics 24.0. Data were downloaded weekly and stored on the research team’s server. Personal data were stored separately in a hard copy folder in the researchers’ office.

Baseline characteristics of the three groups, as well as familiarity, arousal, and valence of all the films, were compared using univariate ANOVAs followed by Bonferroni-adjusted pairwise comparisons. Interaction effects between time of measurement (i.e., measurements of t_2_ and t_3_ for positive and negative affect; measurements of t_1_ and t_3_ for all other outcome variables) and group (i.e., plane crash, Fukushima, Congress) on all the dependent variables (i.e., primary outcome: stereotype awareness, stereotype agreement, self-concurrence, and self-stigmatization, secondary outcome: negative affect, positive affect, and self-esteem) were analysed using general linear models with repeated measures. If interactions in these first analyses were statistically significant, one-way ANOVAs of the change in scores (post-film score minus pre-film score) were conducted, followed by Bonferroni-adjusted pairwise comparisons to identify specific group differences. Effect sizes are reported as partial η^2^ values (0.01 ≤ *η*_*p*_^*2*^ ≤ 0.06 small effect; 0.06 ≤ *η*_*p*_^*2*^ ≤ 0.14 medium effect; *η*_*p*_^*2*^ ≥ 0.14 large effect) [37]. Analyses were run with IBM SPSS Statistics 24.0.

## Results

### Sample characteristics

Sample characteristics of the 180 study participants are presented in Table 1. Fifty percent of the participants were in outpatient and inpatient treatment each. According to the International Classification of Diseases 10 (ICD 10; [38]), treatment diagnoses were recurrent depressive disorder (F33; 60.0%), major depressive disorder (F32; 30.0%), dysthymia (F34.1; 3.3%), and other depressive episodes (6.7%). The average age was 38.8 years, 58.9% were female, 50.0% were single, and 42.8% had a rather high level of education. The groups did not differ significantly regarding age, gender, level of education, current depressive symptoms, and affectivity at baseline.

**Table 1:** Baseline characteristics by group

	Germanwings Plane Crash	Fukushima Tsunami	Depression Day Congress	Group Differences
Sample size	60	60	60	
Age (years)				*F*(2,177) = 2.12, *p* = 0.12
Mean (standard deviation)	38.65 (13.32)	41.22 (12.65)	36.52 (11.55)	
Gender (%)				χ^2^(2, *N* = 180) = 5.69, *p* = 0.06
Male	53.3	33.3	36.7	
Female	46.7	66.7	63.3	
Level of education(%)				χ^2^(2, *N* = 180) = 1.68, *p* = 0.43
< 12 years of school	63.3	56.7	51.7	
≥ 12 years of school	36.7	43.3	48.3	
Current depression: Yes (%)	100	100	100	
PHQ-9				*F*(2,177) = 0.01, *p* = 1.00
Mean (standard deviation)	13.97 (5.44)	13.93 (5.54)	13.87 (6.27)	
Negative Affect (PANAS) (t_1_)				*F*(2,177) = 2.57, *p* = 0.08
Mean (standard deviation)	17.62 (7.36)	15.08 (4.54)	16.47 (6.17)	
Positive Affect (PANAS) (t_1_)				*F*(2,177) = 0.54, *p* = 0.59
Mean (standard deviation)	26.42 (7.83)	25.03 (8.07)	25.84 (7.61)	

*PHQ-9* depression scale of the Patient Health Questionnaire, *PANAS* Positive and Negative Affect Schedule, *t*_*1*_ time of measurement before allocation, time of measurement 1

### Manipulation check

Based on Heydendorff’s and Dressing’s categorical system of risky coverage [11], four separate cases of risky coverage could be identified in the experimental condition, i.e., mental health or depression (attribute) of the co-pilot were mentioned as causally related to the plane crash (crime). Additionally, three explicit stigmatizations could be discerned regarding professional bans for people with mental illness. Neither in CG1 nor in CG2 could any risky coverage or explicit stigmatization be found.

Univariate ANOVAs and Bonferroni-adjusted pairwise comparisons indicated differences in familiarity (F (2,177) = 16.44, *p* < 0.001, *η*_*p*_^*2*^ *=* 0.16), arousal (F (2,177) = 10.30, *p* < 0.001, *η*_*p*_^*2*^ *=* 0.10), and valence (F2,177) = 114.24, *p* < 0.001, *η*_*p*_^*2*^ *=* 0.56) as expected: (i) The Depression Day Congress was less known than the other two topics, (ii) arousal ratings were higher for the plane crash than for the Depression Day Congress, and (iii) valence ratings were more positive than for both EG and CG1.

### Experimental phase: main and interaction effects

Table 2 summarizes the means and standard deviations of outcome variables across groups.

**Table 2:** Summary of means and standard deviations of outcome variables across conditions

Measures		Germanwings Plane Crash						Fukushima Tsunami						Depression Day Congress				
		Pretest			Posttest			Pretest			Posttest			Pretest			Posttest	
		Mean	SD		Mean	SD		Mean	SD		Mean	SD		Mean	SD		Mean	SD
Awareness (SSMIS)		49.85	19.25		52.38	20.20		46.30	16.45		49.93	18.74		44.28	18.78		44.67	21.48
Agreement (SSMIS)		26.82	14.18		32.30	15.75		26.25	13.75		25.92	13.07		20.92	8.84		19.35	8.61
Self-concurrence (SSMIS)		27.65	14.58		26.70	16.26		25.95	12.61		21.77	10.16		24.17	11.25		21.87	11.16
Self-Stigma (SSDS-D)		2.95	0.92		2.89	1.01		3.03	0.96		2.94	0.99		3.09	0.83		2.96	0.91
Shame		2.90	1.24		2.75	1.34		3.03	1.23		2.83	1.27		3.29	1.12		2.94	1.21
Self-Blame		3.42	1.00		3.24	1.11		3.56	0.95		3.39	1.00		3.57	0.95		3.31	1.05
Social Inadequacy		2.99	1.10		3.04	1.15		2.98	1.27		3.03	1.29		3.00	1.11		3.08	1.17
Help-Seeking Inhibition		2.49	0.99		2.54	1.09		2.57	1.04		2.50	1.18		2.52	0.98		2.51	1.09
Self-Esteem		14.57	5.89		14.28	6.98		14.03	6.34		13.97	7.12		13.98	6.39		14.47	7.16
Negative Affect (PANAS)		14.40	6.12		24.75	9.48		12.65	4.14		20.53	7.19		13.23	4.67		14.7	4.96
Positive Affect (PANAS)		24.97	8.74		22.27	6.53		22.23	8.27		20.17	5.61		23.78	7.26		23.35	7.87

*Awareness* stereotype awareness, *Agreement* stereotype agreement, *SSMIS* self-stigma of mental illness scale, *SSDS-D* Self-Stigma of Depression Scale for people with depression, *PANAS* Positive and Negative Affect Schedule

Regarding group x time interaction effects, the results of general linear models and subsequent one-way ANOVAs of change scores were unchanged when controlling for age, gender, and current depressive symptoms. Thus, the uncontrolled analyses of general linear models with repeated measures are presented in Table 3. The main effect of time was not significant for stereotype awareness (F (1,177) = 2.61, *p* = 0.11), stereotype agreement (F (1,177) = 2.94, *p* = 0.09), self-esteem (F (1,177) = 0.03, *p* = 0.19), social inadequacy (F (1,177) = 1.63, *p* = 0.20), and help-seeking inhibition (F (1,177) = 0.10, *p* = 0.76).

**Table 3:** Results of multivariate tests of general linear models with repeated measures for each outcome variable

	df	df (error)	F	*p*	*η*_*p*_^*2*^
Awareness (SSMIS)					
main effect time	1	177	2.61	0.108	0.015
main effect group	2	177	2.31	0.102	0.025
interaction effect time x group	2	177	0.50	0.608	0.006
Agreement (SSMIS)					
main effect time	1	177	2.94	0.088	0.016
main effect group	2	177	9.85	0.000	0.100
interaction effect time x group	2	177	9.74	0.000	0.099
Self-concurrence (SSMIS)					
main effect time	1	177	13.19	0.000	0.069
main effect group	2	177	2.01	0.137	0.022
interaction effect time x group	2	177	1.89	0.154	0.021
Self Stigma (SSDS-D)					
main effect time	1	177	7.97	0.005	0.043
main effect group	2	177	0.20	0.822	0.002
interaction effect time x group	2	177	0.40	0.672	0.004
Shame					
main effect time	1	177	18.97	0.000	0.097
main effect group	2	177	0.924	0.399	0.013
interaction effect time x group	2	177	1.204	0.302	0.013
Self-Blame					
main effect time	1	177	15.94	0.000	0.083
main effect group	2	177	0.35	0.708	0.004
interaction effect time x group	2	177	0.32	0.727	0.004
Social Inadequacy					
main effect time	1	177	1.63	0.204	0.009
main effect group	2	177	0.01	0.987	0.000
interaction effect time x group	2	177	0.02	0.977	0.000
Help-Seeking inhibition					
main effect time	1	177	0.10	0.757	0.001
main effect group	2	177	0.01	0.993	0.000
interaction effect time x group	2	177	0.61	0.546	0.007
Self-Esteem					
main effect time	1	177	0.03	0.863	0.000
main effect group	2	177	0.07	0.937	0.001
interaction effect time x group	2	177	0.79	0.456	0.009
Negative Affect (PANAS)					
main effect time	1	177	192.99	0.000	0.522
main effect group	2	177	15.55	0.000	0.149
interaction effect time x group	2	177	31.37	0.000	0.262
Positive Affect (PANAS)					
main effect time	1	177	14.44	0.000	0.075
main effect group	2	177	2.48	0.087	0.027
interaction effect time x group	2	177	2.19	0.115	0.024

*Awareness* stereotype awareness, *Agreement* stereotype agreement, *SSMIS* self-stigma of mental illness scale, *SSDS-D* Self-Stigma of Depression Scale for people with depression, *PANAS* Positive and Negative Affect Schedule; time of measurements: measurements of t_2_ and t_3_ for positive and negative affect; measurements of t_1_ and t_3_ for all other outcome variables; group: plane crash, Fukushima, congress)

There was a main effect of time for a decrease in self-concussion (F (1,177) = 13.19, *p* < 0.001, *η*_*p*_^*2*^ *=* 0.07) and in self-stigma of depression (F (1,177) = 7.97, *p* < 0.01, *η*_*p*_^*2*^ *=* 0.04), shame ((F (1,177) = 4.96, *p* < 0.001, *η*_*p*_^*2*^ *=* 0.10), self-blame (F (1,177) = 15.94, *p* < 0.001, *η*_*p*_^*2*^ *=* 0.08), and positive affect (F (1,177) = 14.44, *p* < 0.001, *η*_*p*_^*2*^ *=* 0.08) and for an increase in negative affect (F (1,177) = 192.99, *p* < 0.001, *η*_*p*_^*2*^ *=* 0.52).

Most important, there were significant group × time interaction effects for stereotype agreement (*F* (2,177) = 9.74, *p* < 0.001, *η*_*p*_^*2*^ *=* 0.10) and negative affect (*F* (2,177) = 31.37, *p* < 0.001, *η*_*p*_^*2*^ *=* 0.26) but not for any of the remaining measures. In addition, the robustness of these results was confirmed via hierarchical regression analyses.^1^

To identify the interaction effects, subsequent one-way ANOVAs of the change in scores of stereotype agreement and negative effect were conducted. A significant main effect of group was found for both stereotype awareness (F (2,177) = 9.74, *p* < 0.001, *η*_*p*_^*2*^ = 0.10) and negative affect (F (2,177) = 31.37, *p* < 0.001, *η*_*p*_^*2*^ *=* 0.26). Pairwise comparisons revealed the increase in stereotype agreement as significantly higher in EG than in CG1 (mean difference = 5.17, 95% CI = 1.69–9.94, *p* < 0.01) and in CG2 (mean difference = 7.05, 95% CI = 2.93–11.17, *p* < 0.001). There was no significant difference in change between CG1 and CG2 (mean difference = 1.23, 95% CI = − 2.89-5.36, *p* = 1.00). Relative to CG2, the increase in negative affect was significantly higher in EG (mean difference = 8.83, 95% CI = 6.08–11.68, *p* < 0.001) and in CG1 (mean difference = 6.42, 95% CI = 3.62–9.22, *p* < 0.001). There was no significant difference in change in negative affect between EG and CG1 (mean difference = 2.47, 95% CI = − 0.33-5.27, *p* = 0.10).

**Fig. 3:**
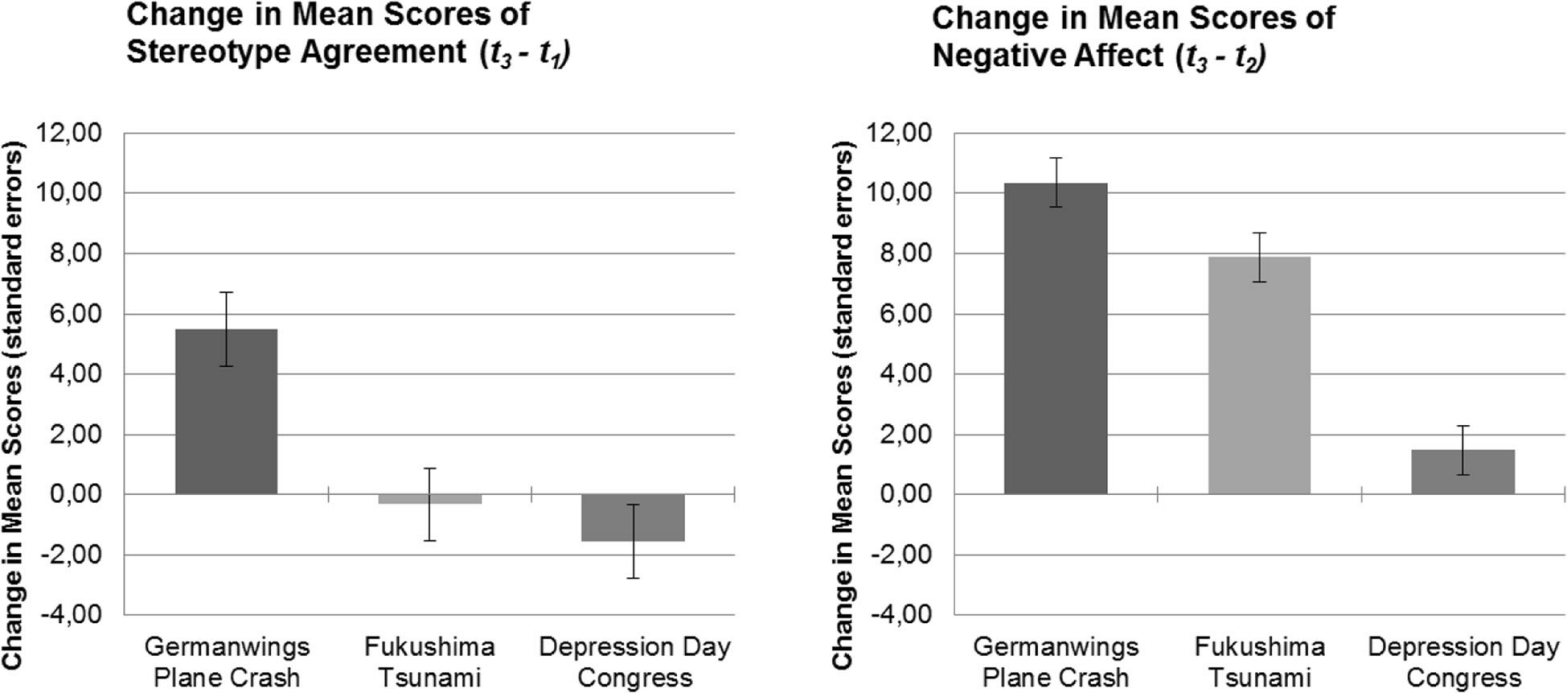
Changes in scores and standard errors for stereotype awareness and negative affect by group. Legend: t_1_ = baseline measurement before watching any films; t_2_ = after watching the nature documentary; t_3_ = after watching the second film

The changes in mean scores and standard errors are shown in Fig. 3 for stereotype agreement and negative affect in each group.

## Discussion

The present study was the first study that aimed at investigating the effect of SMC experimentally on stereotype awareness, stereotype agreement, self-concurrence, self-esteem, and affectivity in persons with the stigmatized attribute, i.e., depression. Hypotheses were based on Corrigan, Rafacz, and Rüsch’s progressive model of self-stigma and tested with an experimental laboratory trial using a controlled design with three parallel randomized groups [19]. In support of the hypotheses, the experimental group indicated higher levels of stereotype agreement and negative affect after watching a film about a negative event referring to depression. Also supporting the hypotheses, watching a film about a negative event without reference to depression resulted in higher levels of negative affect but not in an increase in stigma-measures. Additionally, the effect of a film about a neutral event referring to depression on negative affect was smaller compared to that in the other groups; the effect on stereotype awareness was the reverse of that in the experimental group and did not differ from the condition without relation to depression. The interaction effect for stereotype agreement was of medium effect size and the interaction effect for negative affect of large effect size, both important.

Contrary to the hypotheses, these effects could not be found for other stigma-measures, self-esteem, or positive affect. Kohls and colleagues found corresponding results [39]: In their research, stereotype agreement seemed more easily influenced by an anti-stigma campaign than stereotype-awareness. Regarding the content of the potentially stigmatizing film used in this study, it does not refer directly to the public’s attitudes but rather points out why specific measures should be taken because of potential dangerousness of people with mental illness. The focus is on notional facts of experts but not on what the public in general believes, which might explain a lack of change in stereotype awareness.

The familiarity of the films may also play an important role in stereotype awareness: The Germanwings plane crash was of public interest. Several media reports in print and on television were published over two years, during which public perception could have been formed. Reminding the study participants of this specific event might not have added crucial information about the public’s view and therefore about the participants’ perception about it.

According to Corrigan, the application of stereotype agreement to oneself must meet specific prerequisites [18]: identification with the stigmatized attribute and group as well as the perception of legitimacy of the information received. The study samples consisted of persons carrying not only the stigmatized attribute of mental illness but even more specifically depressive diagnoses. As such, identification with the pilot could be expected to be higher than in a more heterogeneous sample. At the same time, acute suicidality was an exclusion criterion for ethical reasons, namely, protection of participants from serious harm. Most participants might not identify with the characteristics presented in the film and therefore would be protected from applying stigmatizing attitudes to themselves. Scherr and Reinemann indicated comparable findings, as exposure to suicidal media enhances belief in a change in thoughts, perceptions, and behaviour primed by violent media content in other people rather than in oneself [40].

Moreover, protective factors such as emotional clarity [41], cognitive appraisal and a variety of coping responses [42] have been postulated to impact stigmatization effects. Future experiments may investigate potential protective factors, which may buffer the effect between stereotype agreement and self-concurrence or self-stigmatization.

There was a significant decrease over time in self-concurrence, self-stigmatization of depression, shame, self-blame, and positive affect during the experimental phase. These main effects may have arisen as methodological artefact. Schemata of depression and stigmatization were activated by informed consent. This priming may have led to easy accessibility and high values of affectivity and stigma-measures during baseline measurement [43]. As indicated by the adaptation phase, in which both positive and negative affect decreased over time but independently of groups, regression to the mean can be assumed for both stigma- and affectivity measures. Considering regression to the mean – which predicts a decrease in all measures – medium to large interaction effects with increases of negative affect and stereotype agreement in the experimental condition appear particularly important for research and practice.

General strengths of experimental randomized controlled studies can be noted in reference to this study: There were two control groups, in which participants watched films without potentially stigmatizing content but partly about a negative event and partly about the stigmatized attribute. As such, specific stigmatizing effects could be controlled for potential confounding effects of negative affect or activation of depression schemata. Conducting manipulation checks validated intentions of the chosen conditions. Randomization guaranteed a minimization of selection bias. It is notable that no control groups of community samples or participants with other mental illnesses without depressive symptoms were added to the study design. Most measures would have been applicable to patients with mental illness in general. In addition to stereotype awareness and stereotype agreement, hypothetical self-stigma measures could have been used in a community sample. However, research indicated no to very small changes in the community’s attitudes towards persons with mental illness after SMC about the 2015 Germanwings plane crash in France [17]. Adding such a control group to test the null hypothesis requires a very large sample. The added value would have been relatively low for the research questions of interest in this study, especially regarding self-stigma and harm-to-self. However, nothing in the results of this study suggests a specificity of effects for depression. Since the results mainly indicate an impact on stereotype agreement, independently of applying these attitudes to oneself, future research may control for community samples and samples of other mental illnesses to investigate differential effects on different target groups.

The following study limitations should also be addressed in future research: There was no follow-up measurement to determine long-term effects. As such, the present study can only give implications for immediate changes after watching the films. No conclusions can be drawn about persistent changes over days or even weeks.

There is also a lack of information about actual behaviour but rather only self-reported attitudes.

To guarantee that participants met the diagnostic criteria, only patients currently in treatment were recruited to participate. This might have led to selection bias regarding the level of self-stigmatization since help seeking is related to low self-stigma [44].

Another limitation concerns the difference in familiarity of the topics of the films of the conditions. While the Depression Day Congress was not particularly well known, most participants were familiar with the other two events. It could be interesting for future research to examine the effect of SMC covering new instead of familiar information.

There was a trend towards more males in the experimental group. Regression analyses indicated gender and age to have an effect on negative affect. Controlling for gender and age in the general linear models did not change any results regarding the interaction effect. However, these results imply paying attention in future research to such factors as gender or age might moderate the effect of SMC.

### Implications for practice

Confrontation with bad news seems to have a direct stigmatizing effect on people with depression, even if they are familiar with the information. News covering stigmatizing statements about people with mental illness form attitudes of people with depression. At least a short-term increase in stereotype agreement is indicated by the results of this study. Stereotype agreement is an important stage in the process of self-stigmatization, according to the stigma-model of Corrigan and colleagues [19]. Later stages of the self-stigmatizing process, namely, self-concurrence and harm-to-self, were not significantly influenced by watching short films of news at a single point in time. However, time spent reading tabloids was associated with higher endorsement of suicide myths, a lower level of suicide-related knowledge and a higher level of stigmatizing attitudes in recent studies [45]. Therefore, it can be postulated that extensive massive confrontation with SMC over a longer period of time might have a potent effect on self-stigmatization of people with mental illness. One of the most relevant motives for use of traditional media in people suffering under depressive symptoms is information seeking, independently of the depression severity; the higher the levels of depressive symptoms are, the stronger the motivation is to use media as a form of coping to forget about everyday concerns with the intention to overcome depression [46]. It is therefore important to train journalists in reporting in a non-stigmatizing manner about events and at the same time covering information that is relevant in this context. Some events, such as the Germanwings plane crash, may be intrinsically stigmatizing as indications for suicide cannot be denied. Instead of generalizing potential characteristics of the Germanwings pilot to the many people suffering from depression and suicidal ideation, media should rather call attention to how the person involved in this incident differs from these millions of people who do not harm anyone else.

On the other hand, news about the Depression Day Congress, which educates about depression and treatments, did not have a negative effect on affectivity or stigmatizing-processes. Both content and the way of reporting seem to play important roles in this context. For instance, character empathy was indicated as an important mediator for destigmatizing effects of media regarding Paralympic athletes: While pity decreased destigmatization, positive emotions increased destigmatization [47]. A similar effect may be found regarding media about mental illness and should be investigated in the future. Fairmedia is one example of an initiative that guides journalists in reporting about mental illness without stigmatization and discrimination [48].

## Conclusions

Based on the results of this study, specific interventions related to media reporting may be developed for both patients (regarding the reaction to bad news in media and the management of potentially stigmatizing news) and for public prevention (e.g., psychoeducational programmes). Media coverage as a main influencer of public attitudes must further develop and use its power for prevention and education instead of propagating stigmatization of mental illness.

## Additional file

Additional file 1: Trial registration details. (DOCX 15 kb)

## Footnotes

1 Baseline measurements were entered blockwise as independent variables: socio-demographics in step 1 (age, gender, level of education), all variables of relevance in step 2 (PHQ, SSMIS-Subscales, SSDS-D-Subscales, PANAS), and dummy coding for the 3 experimental conditions (i.e., EG = 1, 0, 0; CG1 = 0, 1, 0; CG2 = 0, 0, 1) in step 3.

## Abbreviations

CG1: Control group 1
CG2: Control group 2
CIMH: Central Institute for Mental Health, Medical Faculty Mannheim / University Heidelberg, Germany
EG: Experimental group
PANAS: Positive and Negative Affect Schedule
PHQ-9: Patient Health Questionnaire 9
SMC: Stigmatizing media coverage
SSDS-D: Self-Stigma of Depression Scale for people with depression
SSMIS: Self-stigma of mental illness scale
T_0_, T_1_, T_2_, T_3_: Time of measurement before allocation, time of measurement 1, time of measurement 2, time of measurement 3
