# Supplementary material for: Effects of stigmatizing media coverage on stigma measures, self-esteem, and affectivity in persons with depression – an experimental controlled trial
Source: BMC Psychiatry. 2019 May 7;19:138. doi: 10.1186/s12888-019-2123-6 (PMC6505308; doi:10.1186/s12888-019-2123-6)
Supplement: Supplementary file 1 — Trial registration details. (DOCX 15 kb) [file 12888_2019_2123_MOESM1_ESM.docx]

**Trial registration details**

| **Data Category** | **Information** |
| --- | --- |
| Primary registry and trial identifying number | German Clinical Trials Register  DRKS00011855 |
| Date of registration in primary registry | 23/06/2017, retrospectively |
| Secondary identifying numbers | 2016-655N-MA |
| source(s) of monetary or material support; primary sponsor | Adjunct Prof. Dr. Josef Bailer  Manager of Center of Psychological Psychotherapy Mannheim, Studies and Outpatient Clinic Manager  Department of Clinical Psychology, Central Institute of Mental Health, Medical Faculty Mannheim / University Heidelberg, Germany  J5, 68159 Mannheim |
| contact for public and scientific queries | Mrs. Nele Göpfert  nele.goepfert@zi-mannheim.de |
| public title | Effects of Media Reports on Self-Stigmatization, Self-Esteem and Affectivity in Persons with Depression |
| scientific title | Effects of Media Reports on Self-Stigmatization, Self-Esteem and Affectivity in Persons with Depression - MeStiD |
| countries of recruitment | Germany |
| health condition(s) or problem(s) studied | Depression |
| intervention(s) | Experimental group: Arm 1 - negative event referring to depression  Control group 2: Arm 2 - negative event without reference to depression  Control group 3: Arm 3 - neutral event referring to depression |
| key inclusion and exclusion criteria | Inclusion criteria: minimum of one depressive episode lifetime, sufficient cognitive abilities and German language skills  Exclusion criteria: acute psychotic, manic, or hypomanic episode, acute addiction symptoms, acute suicidal tendencies  Age minimum: 18 Years  Age maximum: 70 Years  Gender: both, male and female |
| study type | Experimental  Allocation: randomized  Intervention model: parallel assignment  Masking: Open (masking not used) |
| date of first enrolment | 06/03/2017 |
| target sample size | 180 |
| recruitment status | completed |
| primary outcome(s) | Degree of self-stigmatization using the German version of Self Stigma of Depression Scale and the German version of Self-Stigma of Mental Illness Scale directly after watching the videos. |
| key secondary outcomes | Degree of self-esteem (Rosenberg, 1965; Collani & Herzberg, 2003) positive and negative affect (PANAS), as well as the reaction to the Videos regarding valence and arousal (SAM; Bradley & Lang, 1994) |
